# From Junior to Elite in Soccer: Exploring the Relative Age Effect and Talent Selection in Spanish Youth National Teams

**DOI:** 10.3390/children9101543

**Published:** 2022-10-10

**Authors:** Javier García-Rubio, Andrés García-Vallejo, María de los Ángeles Arenas-Pareja, Pablo López-Sierra, Sergio J. Ibáñez

**Affiliations:** Optimization of Training and Sports Performance Research Group (GOERD), Faculty of Sports Science, Universidad de Extremadura, 10003 Cáceres, Spain

**Keywords:** talent selection, Relative Age Effect, maturation, performance, soccer

## Abstract

The implications of relative age grouping in sport are known as the Relative Age Effect (RAE). This study has the twofold purpose of analyzing RAE in Spanish youth national soccer teams and examining the prediction value of being selected for national youth teams to be a professional. The sample was composed of 548 players divided into five groups. A descriptive analysis of distribution and participation, frequencies, mean and standard deviation, crosstabs, Sankey charts, coefficient correlation and Cohen’s effect size criteria and two regression analyses were performed. Results established that the RAE is present in U’17 to U’21 Spanish youth national teams. Talent detection and selection programs are more reliable the closer they are to adulthood, reaching a success rate of almost 100% at the U’21 stage. The selection of players for such programs should be delayed as much as possible, thus, preventing younger players from dropping out and those selected from thinking they have already reached their goal. To this end, they should focus on long-term improvement, not short-term performance. In addition, factors such as the RAE or the maturity level of the athletes should be monitored.

## 1. Introduction

Research and analysis of talent detection and development have grown considerably over the last 20 years [1]. This interest continues due to the unreliability and predictability of talent identification programs [2]. In fact, factors such as relative age, growth, maturation or years of training are still not adequately taken into account, focusing mainly on current performance and not on development [3].

In sport, it is very common to group athletes according to their date of birth, either on an annual or biannual basis, to limit large age differences in competitors [4]. Athletes born at the beginning of the grouping date (usually January 1), have an advantage over those born at the end of the grouping date [5,6]. The consequences of this relative age grouping are known as the Relative Age Effect (RAE) [7]. This results in more athletes born in the first half of the year in the lower sport categories, mainly due to maturational aspects such as anthropometric, physiological or physical/conditional factors. In talent detection and selection programs there is a strong influence of the RAE [4,8]. This is explained by the physical and physiological competitive advantages that players born early in the year have compared to those born later in the same year. These players are selected for what they are in the short term, instead of being selected for what they could be in the future [9,10].

Paradoxically, when the senior level is reached this difference between those born in the first half of the year and those born in the second half lessens and even disappears [11,12]. This phenomenon is known as RAE reversal [12]. Players born later in the year, the oldest players, who make it to the high-performance level, have more successful careers in terms of competitive experiences, longevity in the sport, results or salaries [11]. Being born in the second part of the year, with physical disadvantages compared to those born in the first part, means that young players must develop different skills to be able to compete at the same level as older players [4]. These skills are going to allow them to achieve higher performance at the senior stage, as the physical disadvantages disappear, but not the acquired technical–tactical skills [13], in addition to the psychological ones [14].

The RAE is gradually reduced, without disappearing, up until the senior category [15]. That is, the players who were the best in training categories are gradually being matched and surpassed by other players who were not selected in lower categories. The physical advantage they had when beginning the sport, disappears as the other players mature and, in addition, these differences that were very important during the developmental stages, are no longer so important [16]. In fact, other skills that are decisive for achieving high performance, such as a high degree of resilience [17] or the ability to cope with adversity [18], become evident. Another explanation is the smaller number of injuries presented by these players compared to those who have specialized from a young age [19].

Sport specialization is understood as intense training in one sport to the exclusion of others [20]. This approach to sport practice looks to maximize the performance potential of athletes. Coaches, parents and athletes believe that in this way they will acquire specific skills that will allow them to reach the elite level. Ericsson, Krampe and Tesch-Römer [21] popularized the belief that the greater the amount of specific and intense practice in a sport, the higher the performance. Therefore, one must start at an early age to succeed in a sport [22]. Sport specialization is associated in the long term with overuse injuries and abandonment of sport practice due to burnout [23]. Indeed, the most talented players, capable of becoming elite soccer players, should be identified at the right time [24], not as early as possible.

In view of the above, this study has two differentiated objectives: (a) to analyze the participation of players in the different youth categories of the Spanish national soccer team according to relative age, and (b) to determine the relationship between the youth categories and whether the players who participate in them go on to play soccer professionally.

## 2. Materials and Methods

### 2.1. Sample and Population

The population was composed of all the male players who participated, at least once, in national football team competitions, official or friendly matches, in the under 17 (U’17), under 19 (U’19), under 20 (U’20) and under 21 (U’21) categories, and senior national team players born from 1990 to 2005. The sample (*n* = 548) was distributed as follows: U’17 (*n* = 112), U’19 (*n* = 225), U’20 (*n* = 235), U’21 (*n* = 335), senior (*n* = 126). Data were retrieved principally from a specialized webpage of historic and statistical data in soccer (https://www.bdfutbol.com/es/index.html (accessed on 1 March 2020)). In addition, data were checked in other webpages, such as the Spanish football federation webpage, sites of international competitions of each category, sport journals and personal sites.

### 2.2. Measurements

The study analyzes the following variables: chronological age, the birth quarter of players (Q1 (January, February, March), Q2 (April, May, June), Q3 (July, August, September) and Q4 (October, November, December)) [25,26], specific playing position, seasons in first and second soccer division of the country, games played in each division and national team categories (U’17, U’19, U’20, U’21 and senior national team).

### 2.3. Data Analysis

First, a descriptive analysis was carried out of the sample distribution and participation in national teams. Different analyses were used according to data nature, frequencies, mean and standard deviation, crosstabs and Sankey charts. The percentages of players who have reached the professional category were also used. The coefficient of correlation was used to identify the participation in different national teams and professional soccer. The statistical significance of Pearson′s correlation coefficients depends on sample size, so effect sizes of correlations were reported because of varied sample size, and Cohen’s effect size criteria for correlation coefficients was used to interpret them (small: |r| = 0.10–0.29, medium: |r| = 0.30–0.49 and large: |r| = 0.50 [27]). Finally, two regression analyses were carried out to predict the possibilities of being a professional soccer player and participating in the senior national team. Participation in different youth national teams was analyzed to explore its effects on final performance, identified as games played in the first division. The Durbin-Watson test was used to check whether the residuals in the model were independent and look within the data to control collinearity effects. A binary logistic regression was used to predict national team participation in the function of youth national games participation. Four independent dichotomic variables were included in the model: participation in U’17, U’18, U’19 and U’21 youth national teams. The dependent variable used in this model was Y [0, 1]. The values of the dependent variable were 1 for participation in the national team and 0 for players that had not participated in national teams. For this model, the odds ratios (ORs) and a 95% confidence interval (CI) were determined. The ORs explain the increased odds of the outcome occuring. If the value is greater than 1, then the odds are bigger. The statistical analyses were performed using SPSS v.21 software (Inc., Chicago, IL, USA). Statistical significance was set at *p* < 0.05.

## 3. Results

Table 1 shows how births were distributed across the year in all the analyzed categories. In all cases, the first trimester was when the most births occurred, and the first half of the year establishes that the Relative Age Effect is present in soccer and lasts in all youth categories in Spanish national teams.

**Figure 1:**
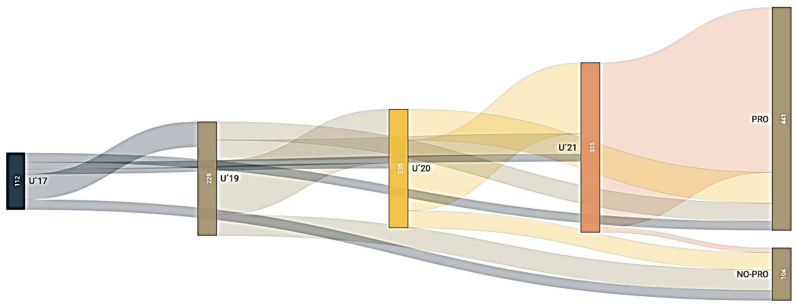
Players’ careers across national teams.

Figure 1 shows how players’ careers developed through national teams before achieving professional status. The Sankey diagram shows how each player’s career develops over time.

The figure indicates the path that the selected players follow until they reach the professional soccer league or not. Among the players called up for the U’17 national team (*n* = 112), there were 82 players who became professionals, but only 13 players went through all the lower national teams until they became professionals. In addition to the 50 players who went on to U’19, there were another 173 players who were selected for the first time. Of these, 167 made it to the professional level, but only 107 were selected at the senior level. Of the 335 players in the U’21 category, 194 made it to the professional level, some through the senior category (*n* = 140) and others directly (*n* = 54). Of the 335 players selected in the U’21, there were nine who did not become professionals in the first division.

**Figure 2:**
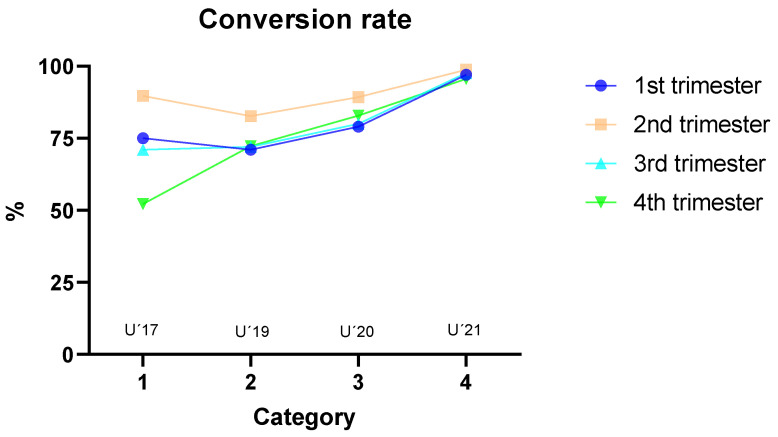
Percentage of players that achieve the professional level according to birth trimester.

Table 2 shows descriptive statistics (number and percentage) of players’ careers till their debut or not in first division soccer. It can be observed that the U’21 category is the most prolific compared with the U’17 or U’19 categories. Figure 2 shows conversion rate to 1^st^ division in each national team according to birth trimester.

Table 3 shows correlation coefficients among different Spanish youth national team players and their first division debut. The U’21 national team is the only category that has a positive impact on being a top professional. Whereas, the U’17 and U’19 national teams have a negative impact.

Lastly, Table 4 shows a logistic regression among all categories. In line with the previous results, being a member of the U’21 national team predicts being a professional in top leagues (R^2^ = 0.40). In Table 5, a linear regression shows identical results when predicting games played at top-level soccer. Only the U’21 national team had a positive influence on later performance (R^2^ = 0.40).

## 4. Discussion

The objective of this study was twofold: (a) to analyze the RAE in Spanish youth national teams and (b) to study the prediction value of being selected for national youth teams for being a professional. The results established that the RAE is present in U’17 to U’21 Spanish youth national teams. In addition, the U’21 national team is the only one that predicts professional performance. Talent detection and development programs have a success rate of about 30% [28]. Usually, these processes focus on successful athletes who have excelled at an early age, using short-term performance indicators such as physical or anthropometric parameters. The RAE notably influences these, leaving aside others such as decision-making, perception, etc., [29]. On the other hand, it is known that relatively younger athletes perform better when they reach adulthood [30].

The RAE has previously been studied in sports [4,5,14] with different effects and sizes according to age or type of sport. The literature suggests that this effect tends to disappear with age [31]. This study sample was selected to begin with 17-year-old players, skipping early development stages, when the RAE is more solid. In sports such as soccer, physical maturation influences physical conditions like strength, endurance and speed [12], the key to sporting success. These characteristics allow older players to gain an advantage over their peers [32]. In addition, differences in the maturational development of young players are going to negatively affect talent identification and player selection [15]. Relatively older players are going to enjoy more opportunities to play, better coaches and competitions, which is going to make the gap between those born earlier and later widen. Around 25% of the U’17 and U’19 players analyzed in this study do not become professionals. Aspects such as date of birth must be taken into consideration when selecting players [33]. Current proposals, such as organized bio-banding competitions, will allow talent detection to be based on technical–tactical issues and not on pure maturation.

Paradoxically, it has been previously shown in basketball that being selected for the junior teams of the national basketball team had a negative impact on players’ subsequent development [34]. In fact, being selected at these ages does not predict high performance [35]. These results support the findings of this study. Participating in the lower national team categories does not have a positive impact on the final development of young soccer players. In these national teams, players are chosen for what they can do in this moment, seeking performance over other characteristics. When the physical advantages at those ages are matched by the rest of their peers, the skills that others have had to develop to be at the same level, are going to make that initial advantage disappear [13]. In addition, the pursuit of early performance is associated with increased dropout when athletes are under high levels of pressure [36]. This early specialization, in some cases, has a positive influence on subsequent performance. In the case of swimming, junior swimmers who go on to compete at the international level as seniors achieve good results [37].

In fact, clubs select players born earlier in the year for their physical and anthropometric power [38]. This is going to cause the selected boys and girls to dedicate many more hours to sport-specific practice, a highly structured practice known as deliberate practice and what Ericsson [21] points out as fundamental to achieving excellence. This initial push in sport expertise means that the rest of the motor experiences or sport skills are scarce, limiting motor development [39]. It seems clear that the soccer players in this study have undergone an early specialization to become international players in training categories at 16 or 17 years of age which has limited their future development and participation in professional competitions.

## 5. Conclusions

Talent detection and selection programs are more reliable the closer they are to adulthood, reaching a success rate of almost 100% at the U’21 stage. The earlier these programs are carried out, the fewer the selected players will arrive at the professional level. In addition, the training processes of talented athletes are complex and multifactorial, so the decisions made must help players stay in the sport. For example, these TID (talent identification and development) programs are strongly influenced by the RAE, which can cause unselected athletes to drop out of the sport.

### Practical Applications

The selection of players for talent programs should be delayed as much as possible, thus, avoiding younger players dropping out and those selected thinking that they have already achieved their goal. TID programs should be focused on long-term improvement, not short-term performance. In addition, factors such as the RAE or the maturity level of the athletes should be monitored.

## Figures and Tables

**Table 1:** Birth distribution according to trimester.

	17	19	20	21	TOTAL
1º	28.6% (32)	33.8% (76)	35.7% (84)	30.4% (102)	32.4% (294)
2º	25.9% (29)	23.1% (52)	23.8% (56)	24.2% (81)	24.0% (218)
3º	25.0% (28)	22.2% (50)	25.5% (60)	25.1% (84)	24.4% (222)
4º	20.5% (23)	20.9% (47)	14.9% (35)	20.3% (68)	19.0% (173)
TOTAL	112	225	235	335	907

**Table 2:** Descriptive statistics of players’ development according to age.

	17	19	20	21
No debut	30	58	41	9
	26.80%	25.80%	17.40%	2.70%
Debut	82	167	194	326
	73.20%	74.20%	82.60%	97.30%

**Table 3:** Correlation Coefficient of different categories of Spanish national teams and first division soccer.

	U’19	U’20		U’21		1st Division	
U’17	0.037	−0.147	**	−0.171	**	−0.101	*
U’19		0.79		−0.202	**	−0.145	**
U’20				−0.028		0.034	
U’21						0.521	**

* *p* < 0.05; ** *p* < 0.01.

**Table 4:** Logistic regression between Spanish national youth teams and playing in first division soccer.

	B	S.E.	Wald	*p*	OR	OR (95% IC)	
						Lower	Upper
U’17	0.038	0.315	0.015	0.903	1.039	0.561	1.925
U’19	0.232	0.273	0.721	0.396	1.261	0.738	2.156
U’20	−0.289	0.282	1.055	0.304	0.749	0.431	1.3
U’21	−3.337	0.374	79.452	0.000	0.036	0.017	0.074
Constant	−0.226	0.3	0.568	0.451	0.798		

R^2^ = 0.40 (Nagelkerke). *p* < 0.001.

**Table 5:** Linear regression between Spanish national youth teams and football games played in the first division.

	B(SD)
Constant	2.39 (0.42)
U’17	−0.23(0.46)
U’19	−0.09(0.38)
U’20	0.26(0.37)
U’21	7.2(0.39) **
R^2^	0.40
Durbin-Watson	1.83
N	548

** *p* < 0.001.
